# Utilisation of national community-based blood pressure monitoring service among adult Chinese and its association with hypertension treatment and blood pressure control—a mediation analysis

**DOI:** 10.1186/s12877-019-1176-1

**Published:** 2019-06-10

**Authors:** Hongxun Song, Donglan Zhang, Zhuo Chen, Ruoxi Wang, Shangfeng Tang, Ghose Bishwajit, Shanquan Chen, Da Feng, Tailai Wu, Yang Wang, Yanwei Su, Zhanchun Feng

**Affiliations:** 1 School of Medicine and Health Management, Tongji Medical College, Huazhong University of Science & Technology, 13 Hangkong Road, Wuhan, Hubei China; 2 College of Public Health, University of Georgia, 305B Wright Hall, Health Sciences Campus, 100 Foster Road, Athens, Georgia; 3 School of International Development and Global Studies, University of Ottawa, 75 Laurier Avenue East, Ottawa, ON Canada; 4 Jockey Club School of Public Health and Primary Care, Chinese University of Hong Kong, Sha Tin, N.T, Hong Kong, SAR China; 5 School of Pharmacy, Tongji Medical College, Huazhong University of Science & Technology, 13 Hangkong Road, Wuhan, Hubei China; 6 School of Nursing, Tongji Medical College, Huazhong University of Science & Technology, 13 Hangkong Road, Wuhan, Hubei China

**Keywords:** BP monitoring, BP control, Medication, Lifestyle behaviours, Chinese, Utilisation

## Abstract

**Background:**

Community-based blood pressure (BP) monitoring plays an important role in national hypertension management in China. However, the utilisation of this service, together with its associations on hypertension treatment and BP control has not been fully investigated.

**Methods:**

The study population was from the China Health and Retirement Longitudinal Study (CHARLS) in 2015. Cross-sectional data of 2487 hypertensive persons were included as subjects. Stratified sample households were selected from 450 villages or communities of 150 counties from 28 provinces. Finally, 21,097 individuals were interviewed successfully. The main outcome was hypertension control (having average BP under 140-90 mmHg). The main independent variable was utilisation of community-based BP monitoring service (having BP examination once a season or more). The mediators were hypertension treatment (currently taking any antihypertensive medicine) and lifestyle factors (alcohol intake, physical activity, smoke). We performed chi-square and binary logistic regression to analyse associations of BP monitoring with hypertension treatment and blood pressure control. The mediation model was examined by the Sobel test.

**Results:**

Mean age of the population was 64.2 (0.24). The percentage of males was 42.8%. Prevalence of community-based BP monitoring was 32.1%. Patients who used this service had higher odds of hypertension treatment (β = 1.259, *P* < 0.01, OR = 3.52, CI = 2.467–5.030), and BP control (β = 0.220, *P* < 0.05, OR = 1.246, CI = 1.035–1.499). Medication treatment played a complete mediating role between monitoring and hypertension control in this study (t = 4.51, *P* < 0.001). Those who underwent BP monitoring tended to be those who did not finish primary school education (χ2 = 30.300, *P* < 0.001), had poorer household income (χ2 = 18.298, *P* < 0.05), and lived in rural areas rather than in urban areas (χ2 = 40.369, *P* < 0.001).

**Conclusions:**

Although the use of BP monitoring service had no direct effect on BP control, it had a positive effect on BP control through the full mediation effect of hypertension treatment. Termly BP monitoring by community-based health expertise among hypertensive persons, for instance, once a season, can be recommended to public health policymakers for BP control through instructions on medication treatment and health behaviours.

## Background

Hypertension is one of the major non-communicable diseases (NCDs) in the world that defined as one’s systolic blood pressure (SBP) above 140 mmHg or diastolic blood pressure (DBP) above 90 mmHg without taking any antihypertensive medicine. Hypertension is associated with higher risks of cardiovascular diseases (CVD) accounting for 17 million deaths in 2013 worldwide [1]. Additionally, It results in other NCDs such as diabetes and chronic kidney diseases. The prevalence of hypertension has been rising in decades in both developed and developing countries [2–7]. In 2010, it was estimated that 31.1% of the world’s adults had hypertension; 28.5% in high-income countries and 31.5% in low-and-middle-income countries [8]. In China, hypertension prevalence is rising as the population grows older. According to a recent study, the prevalence of hypertension among people in the age group of 35–75 is 44.7, 92.6% of whom were above 45 years old [9].

Blood pressure of hypertensive persons is controlled if BP < 140-90 mmHg with or without medication. The major hypertension treatment is to take antihypertensive medicine. As the main cause of uncontrolled BP, hypertension is lack of treatment in most countries. Burden of the hypertension management was severe in many countries including China. A study across four Middle Eastern countries (Iran, Occupied Palestinian Territory, Saudi Arabia, and the United Arab Emirates) presented that 47% of persons with hypertension were treated and only 19% had controlled BP [10]. The study in South Asia showed hypertension treatment was 31.9% and BP of 12.9% was under control. In India, among all persons with hypertension, only 30.1% were known or on treatment, among whom nearly 61% had controlled BP [11]. In Nepal, only 29% of hypertensive persons were receiving treatment, and 8.2% had controlled BP [12]. The burden of hypertension treatment and BP control was even heavier in Africa. In Zambia, only 18.0% had any antihypertensive drug prescribed [13]. In Tanzania, nearly all patients (95.3%) had uncontrolled BP [14]. The condition is better in developed countries. Results of the German Health Examination Survey showed among persons aware of their hypertensive status 37.9% were BP uncontrolled. Among that 33.4% were untreated [15]. In China, hypertension treatment rates were reported under 50%, and BP control rates were lower than 20% [16–18]. In general, when the rate of hypertension treatment is higher, the rate of BP control is higher, too.

In addition to inadequate medication treatment, uncontrolled hypertension is also attributed to unhealthy lifestyle behaviours including smoking, drinking and lack of exercise. In a study identified latent classes of hypertensive persons’ lifestyle risk factors including physical activity, smoking habits, and blood pressure control, about 85% of hypertensive persons were categorized in an intermediate to high-risk class of lifestyle [19]. In China, compared with all hypertensive persons, lower prevalence of smoking (25% vs 28%) and alcohol intake (28% vs 32%) was found among BP controlled persons while prevalence of adequate physical activity was higher (32% vs 25%). But all of them were higher among persons who were being treated (44% vs 28, 49% vs 32, 57% vs 25%) [20–23]. A population survey in Japan stated that prevalence of current smoking and physical activity was 10 and 38.1%, and average alcohol intake was 67.4 g/week among treated hypertensive persons. It was close to the prevalence of total hypertensive population [24]. The condition of unhealthy lifestyles among hypertensive persons seems to be better in the developed country.

Conventionally, hypertensive persons are encouraged to control the symptom by long-term BP monitoring, medication use as well as establishing healthy lifestyle behaviours [25]. Interventions of community-based hypertension treatment have been proved to be effective in BP control. A study in Cameroon showed that BP control was better in those who were adherent to medication (47.5% versus 8.2%) [26]. A significant BP decrease was found after hypertension treatment management of a self-funded group in a resource-poor community in rural Honduras [27]. Improved behaviours are also key to achieve better BP control. Smoking has been reported as one of the most common predictors of uncontrolled blood pressure among hypertensive persons [21]. A study in Uganda indicated that significantly decreased odds of raised blood pressure were associated with moderate-to-vigorous physical activity [28]. Community-oriented hypertension management including treatment components and health promotion was proved to be a feasible way to achieved improvement in medication treatment, behaviours and reduction in BP and CVD risk [29]. However, the standards of community-based intervention were various in these study and the subjects were limited to certain communities. A design concerned of the mechanism of major interventions to a large population is absence. Evaluation of national intervention programs remains a challenge due to the difficulty of linking the program attendance and outcome at the population level.

Utilisation of community-based BP monitoring service was defined as receiving free blood pressure examination at least 4 times annually in the study. In 2009, a national program, Essential Public Health Services (EPHS) was launched in China as part of the new Health Care System Reform [30]. Chronic disease screening and management were included as part of the public health services and provided to community-dwelling patients free of charge, with an aim to attain universal coverage with equitable basic health care. Grass-root health institutions of urban community centres and rural township hospitals are responsible for establishing a health record and for providing free health examinations including BP measurement once a season for every hypertensive person living in their catchment areas. The use of this service at least 4 times a year were defined as utilisation of community-based BP monitoring service. Through monitoring of BP condition, community or village doctors should give instructions on hypertension treatment and health-related life behaviours after checking symptoms [31]. Only a few studies have estimated utilisation of this service since national data on the frequency of community-based BP examinations is scarce. A study showed 8.1% of hypertension patients received the EPHS-covered service by 2013 based on free physical examination paid by the government and it brought about an increase of monitoring, treatment, and control of hypertension [32]. But the evidence is limited on the relationship between community-based BP monitoring with hypertension control. Mechanisms of mediation effect of hypertension treatment and healthy lifestyle behaviours were not considered. It is necessary to examine the utilisation of this service and to find how to improve effectiveness of this program.

**Fig. 1:**
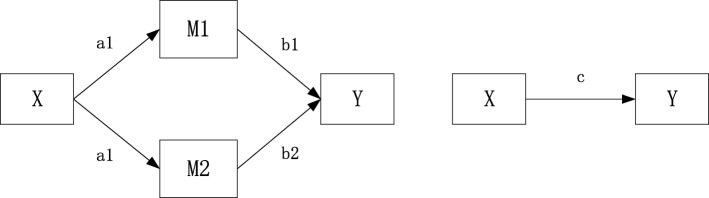
A mediation model of hypothesis**.** X for predictor (community-based BP monitoring), M1, M2 for mediators (hypertension treatment and lifestyle behaviors) and Y for outcome (hypertension control). M1 and M2 are considered as mediators to the extent to which it carries the influence of a given independent variable (X) to a given dependent variable (Y). a1, a2, b1, b2 for indirect effects, c for direct effect

It is believed that patients could achieve a better outcome of BP control if they follow health expertise advice on hypertension treatment and healthy lifestyles in terms of exercising properly [33], avoiding smoking and drinking [34]. Supported by previous study reports, our hypothesis is that community-based BP monitoring has a positive effect on hypertension control through medication treatment and healthy lifestyle establishment. (Fig. 1) Accordingly, this study aims to provide an overall estimate of national-level utilisation of community-based BP monitoring service among hypertensive persons and to explore its association with blood pressure control. Mediation analysis was designed to explore mechanisms of mediation effects of hypertension treatment and healthy lifestyles between them.

## Methods

### Study design

This is a cross-sectional study based on data from the China Health and Retirement Longitudinal Study (CHARLS) 2015. This study is designed to explore the association of community-based BP monitoring utilisation with hypertension control. CHARLS respondents were followed using a computer-aided personal interview every 2 years. A structured questionnaire with several main sections was used to collect data from each subject. One section was used to record height, weight and blood pressure. Each subject’s systolic and diastolic blood pressures were recorded three times by a trained nurse using a HEM-7112 electronic monitor (Omron, Kyoto, Japan). The mean values for each subject were then calculated. The interviewees were asked face-to-face if they were taking any form of anti-hypertensive medication and how often they had blood pressure examination by village or community doctors in the last year.

### Study setting

CHARLS is a nationally representative survey of individuals over age 45 in China. The 2015 survey was the fourth to be conducted in the country. Information about community-based hypertension management was only provided in the recent investigation of this wave. Stratified sample households were selected in 450 villages or communities of 150 counties from 28 provinces. In total, 12,241 households were selected for the survey. 21,097 individuals were interviewed successfully. The de-identified database was publicly available online.

### Study subjects

The members of the selected households aged over 45 years old were chosen as subjects. If a selected household had more than one member aged 45 years or older, one such member – randomly chosen – and his or her spouse if also aged 45 years or older were selected as subjects of the survey. Overall, 21,097 respondents were interviewed in 2015. Subjects were selected for this study if they were identified as hypertensive and provided information about community-based BP monitoring.

### Study period

This study is based on a secondary data analysis. The primary data collection of China Health and Retirement Longitudinal Study (CHARLS) was conducted in 2015. The data request of 2015 CHARLS was approved in May 2018. The study sample selection was conducted from May to August 2018. Data analysis was accomplished from September to October 2018.

### Sampling method

A four-stage, stratified, cluster sampling was used to select primary participants in the baseline CHARLS survey 2011. In the first stage, 150 county-level units from 28 provinces were selected to give a mix of urban and rural settings and a wide variation in the level of economic development. Three primary sampling units – administrative villages in rural areas or communities in urban areas – were then chosen in each selected county-level unit. All of the dwellings in each selected primary sampling unit were then outlined on Google Earth maps using the “CHARLS-GIS” software package that was specifically designed for the survey. Finally, 24 of the mapped households in each primary sampling unit were randomly selected.

In this study, the eligible sample was selected from subjects who had provided BP measures (*n* = 15,247). A subject was considered hypertensive if he or she had a mean systolic blood pressure of ≥140 mmHg, a mean diastolic blood pressure of ≥90 mmHg or was already taking anti-hypertensive medication. Among all subjects provided blood pressure biomarker, 5884 respondents were identified with hypertension, and 3498 of them provided information about community-based BP monitoring. 2487 of subjects provided complete data for this study.

### Selection criteria

Inclusion criteria: Subjects were included if blood pressure biomarker provided (n = 15,247) and identified as hypertensive (*n* = 5884).

Exclusion criteria: Subjects were excluded due to missing values of biomarker (*n* = 5050), missing values of whether who used community-based monitoring service (*n* = 2386), diagnosed comorbidities of stroke (*n* = 268) or chronic kidney diseases (*n* = 456), and missing values of comorbidities (*n* = 661).

### Measurement

The variable of BP control was chosen as the dependent variable. Hypertensive persons with SBP (systolic blood pressure) under 140 mmHg and DBP (diastolic blood pressure) under 90 mmHg were considered to be controlling their hypertension well. According to it, the variable of BP control was categorized by yes and no.

Mediators included one variable of hypertension treatment and three variables of lifestyle behaviours. Hypertensive persons who claimed to be receiving any form of anti-hypertensive medication were considered to be treated. Hypertension treatment was categorized by yes (for those who are taking traditional or western medicine), and no (for those who are not taking any medicine). Three lifestyle behaviours related variables included alcohol intake, physical activity level and smoking. Alcohol intake was categorized by often (drink more than once a month), occasionally (drink less than once a month) and never (never drink) according to the question “How frequent do you drink?” Physical activity level was divided as plenty level (more than 300 min moderate exercise or 150 min vigorous exercise weekly), adequate level (150–300 min moderate exercise or 75–150 min vigorous exercise weekly), light level (less than 150 min moderate exercise or 75 min vigorous exercise weekly) and no exercise at all. Smokers were categorized by yes and no according to the question “Do you currently smoke?”

The independent variable of interest was utilisation of community-based BP monitoring service. Subjects were identified as BP monitored if they received EPHS-covered BP examination once a season (annually 4 times). The variable was categorized by yes (those who had BP examination once a season or more) and no (those who had BP examination less than once a season) according to their answers to the question “How often did you have blood pressure examination by community/village doctors?”

Covariates were respondents’ sociodemographic characteristics, including age (45–59, 60–69, 70–102), sex, educational attainment (no education, elementary, middle school, high school and above), annual household income (poor, near poor, middle income, high income), administrative registration status (rural, urban), cohabitation status (living with spouse or cohabitated), insurance status, BMI (body mass index) and diagnosed chronic diseases. The four groups of household income categories were defined based on the 25th, 50th, and 75th percentiles of annual household income of the subjects. The rural or urban administrative registration status is based on the National Bureau of Statistics definition where a primary sampling units is defined as urban if it is located in a city, suburb of a city, a town, suburb of a town, or other special areas where nonfarm employment constitutes at least 70% of the workforce, such as a special economic zone, state-owned farm enterprise. BMI was defined as weight (kg) divided by height^2^ (m^2^). Subjects who had previously received a doctor’s diagnosis of diabetes, cancer, stomach diseases, or arthritis in CHARLS were considered with diagnosed chronic diseases.

### Data analysis

Socio-demographic characteristics were performed as percentages. A complex sampling plan was constructed according to stratification, cluster, and weights of this survey for chi-square test and binary logistic regression. The Chi-square test was used to compare socio-demographic characteristics, 4 mediators, and hypertension control between respondents monitored by community-based BP examination and those who did not. We also used Chi-square test to examine BP control for independent and mediation variables. To examine the association of BP monitoring with BP control and significant mediators in Chi-square test, we used logistic regression which can take hypertension treatment into consideration. The Sobel test was conducted to examine the mediation effect of medication treatment between independent BP monitoring variable and dependent BP control variable with their coefficients of three regression models above. All models were adjusted for age, gender, educational attainment, household income, cohabitation status, residential region, working status, insurance status, BMI, and chronic diseases diagnosis. Statistical analysis was implemented by using SPSS 13.0 (SPSS Inc., USA).

## Results

Table 1 shows among 2487 hypertensive persons, 69% of them were the elderly. The mean age was 64.2 (SE = 0.24) years old (ranged from 45 to 102). Only 32.1% of subjects had BP monitored by the community or village doctors. 28.0% of subjects were uneducated. 80.0% of them lived with a spouse or cohabitated. 52.8% of them were from rural areas and 54% had a job. 94% of them were insured. 77.9% of hypertensive persons seldom drank. One-fifth of them did not exercise. The prevalence of smoking was 22.8%. The rate of patients taking antihypertensive medicine were 88.1%. Hypertensive persons had their BP monitored in the community or village were more likely to be female, with less household income, with lower level education and from rural areas. It indicated the underuse of community service by richer, higher educated, and urban patients. The hypertension treatment rate was significantly higher in patients who had BP monitored by the community or village doctors.

**Table 1:** Sociodemographic, lifestyles, and hypertension treatment among hypertensive adults and the percentage of patients reported using community-based BP monitoring service, China Health and Retirement Longitudinal Study 2015

Variable	Category	Total	BP monitoring		χ2	*P*
			Yes	No		
Sex					10.412	0.002**>
	Male	42.78%	38.10%	45.00%		
	Female	57.22%	61.90%	55.00%		
Age					1.557	0.537
	45–59	30.62%	31.41%	30.24%		
	60–69	40.20%	41.07%	39.79%		
	70–102	29.18%	27.52%	29.97%		
Education					30.300	< 0.001**
	Uneducated	27.92%	33.06%	25.48%		
	Primary school	38.96%	40.96%	38.02%		
	Middle school	20.52%	16.44%	22.45%		
	High school and above	12.60%	9.54%	14.05%		
Cohabitated					3.006	0.136
	Yes	79.98%	77.95%	80.95%		
	No	20.01%	22.05%	19.05%		
Household income					18.298	0.003**
	Poor	22.91%	24.56%	22.13%		
	Near poor	24.88%	29.22%	22.82%		
	Middle income	26.85%	24.07%	28.17%		
	High income	25.36%	22.15%	26.87%		
Urbanity					40.369	< 0.001**
	Rural	52.78%	62.08%	48.39%		
	Urban	47.22%	37.92%	51.61%		
Currently working					7.253	0.012
	Yes	52.40%	56.34%	50.54%		
	No	47.60%	43.66%	49.46%		
Insured		93.45%	95.43%	92.51%	7.502	0.018*
Alcohol intake					2.991	0.244
	Never	68.44%	70.70%	67.37%		
	Occasionally	8.44%	8.19%	8.56%		
	Often	23.12%	21.11%	24.07%		
Physical Activity					11.352	0.047
	No exercise	20.42%	18.20%	21.47%		
	Light level	34.41%	36.81%	33.28%		
	Adequate level	11.09%	8.87%	12.14%		
	Plenty level	34.08%	36.13%	33.11%		
Smoker					0.054	0.838
	Yes	22.80%	23.09%	22.67%		
	No	77.20%	76.91%	77.33%		
Treatment					54.228	< 0.001**
	Yes	88.04%	95.05%	84.73%		
	No	11.96%	4.95%	15.27%		

*P < 0.05, **P < 0.01

Table 2 presented that 57.4% of subjects had controlled BP. The BP control rate was higher in subjects who had BP monitored and who were treated by hypertensive medication. We found no difference of lifestyles including drinking, smoking, and physical activity in BP control so they were excluded in the further mediation analysis.

**Table 2:** Hypertension control across community-based BP monitoring utilisation, lifestyles, hypertension treatment, China Health and Retirement Longitudinal Study 2015

Variable	Category	Total	Hypertension control		χ2	*P*
			Yes	No		
BP monitoring					6.227	0.014*
Yes		32.11%	34.85%	30.10%		
No		67.89%	65.15%	69.90%		
Alcohol intake					4.116	0.218
Never		68.44%	69.11%	67.95%		
Occasionally		8.44%	9.39%	7.75%		
Often		23.12%	21.49%	24.31%		
Physical Activity					0.386	0.959
No exercise		20.42%	20.56%	20.32%		
Light level		34.41%	34.41%	34.42%		
Adequate level		11.09%	10.64%	11.41%		
Plenty level		34.08%	34.39%	33.85%		
Smoker					0.230	0.696
Yes		22.80%	22.33%	23.15%		
No		77.2%	77.67%	76.85%		
Treatment					242.314	< 0.001**
Yes		88.04%	99.93%	79.32%		
No		11.96%	0.07%	20.68%		

*P < 0.05, **P < 0.01

**Fig. 2:**
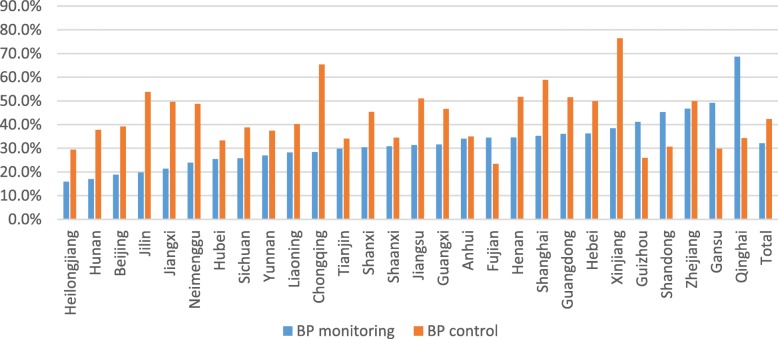
Community-based BP monitoring and BP control of 28 provinces in China

The reach of the community-based BP monitoring service varied largely between different regions (from 16 to 69%), as well as the BP control rate (from 23 to 76%). Among 28 provinces of China, the percentage of BP monitoring patients was the largest in Qinghai and Gansu provinces in western China, while the smallest in Heilongjiang and Hunan province in the central and eastern China. The rate of BP control was the highest in the west of Xinjiang and Chongqing provinces and the lowest in the Fujian and Guizhou Province (Fig. 2).

A binary regression analysis was conducted to investigate factors affected the hypertension treatment. Null hypothesis was β_i_ = 0. The results were shown in Table 3. It indicated significant factors affected hypertension treatment. The hypertension treatment was significantly associated with community-based BP monitoring of hypertensive persons (β = 1.259, *P* < 0.01, OR = 3.52, CI = 2.467–5.030). Patients who were more likely to use antihypertensive medicine tended to be females, living with spouse or cohabitated and with diabetes.

**Table 3:** Binary logistic regression analysis for the mediator of hypertension treatment associated with BP monitoring (Model 1), China Health and Retirement Longitudinal Study 2015

Variable	Category	%	β	*P*	OR	95% CI	
						Upper	Lower
BP monitoring				< 0.001**			
	No(ref)	67.89	1		1		
	Yes	32.11	1.259		3.523	2.467	5.030
Cohabitated				0.010**			
	No(ref)	20.01	1		1		
	Yes	79.98	0.487		1.628	1.123	2.361
Sex				0.002**			
	Male(ref)	42.78	1		1		
	Female	57.22	0.484		1.622	1.201	2.191
Diabetes				0.002**			
	Yes (ref)	18.45	1		1		
	No	81.55	−0.779		0.461	0.285	0.745

OR = Odds Ratio. 95% CI = 95% Confidence interval for odds ratio

Adjusted for age, gender, educational attainment, household income, cohabitation status, residential region, working status, insurance status, BMI, and chronic diseases diagnosis. Wald F = 6.085, P < 0.001

**P < 0.01

A binary logistic regression was conducted to investigate factors affected BP control. As shown in Table 3, BP control was significantly associated with community-based BP monitoring (β = 0.220, *P* < 0.05, OR = 1.246, CI = 1.035–1.499). Subjects more likely to control their BP tended to be female and with higher level education (Table 4).

**Table 4:** Binary logistic regression analysis for BP control associated with community-based BP monitoring service utilisation (Model 2), China Health and Retirement Longitudinal Study 2015

Variable	Category	%	β	*P*	OR	95% CI	
						Upper	Lower
BP monitoring				0.020*			
	No(ref)	67.89	1		1		
	Yes	32.11	0.220		1.246	1.035	1.499
Education				0.029*			
	Uneducated(ref)	27.92	1		1		
	Primary school	38.96	0.334		1.397	1.085	1.800
	Middle school	20.52	0.452		1.571	1.145	2.157
	High school and above	12.60	0.363		1.438	0.969	2.133
Gender				0.034**			
	Male(ref)	42.78	1		1		
	Female	57.22	0.251		1.285	1.019	1.620

OR = Odds Ratio. 95% CI = 95% Confidence interval for odds ratio

Adjusted for age, gender, educational attainment, household income, cohabitation status, residential region, working status, insurance status, BMI, and chronic diseases diagnosis. Wald F = 1.916, P < 0.01

*P < 0.05, **P < 0.01

**Fig. 3:**
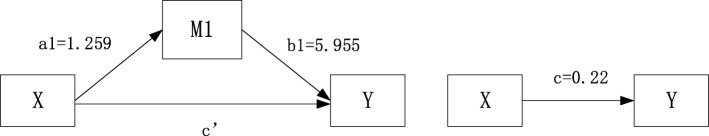
Adjusted mediation model for mediation analysis. X for predictor (community-based BP monitoring), M1 for mediator (hypertension treatment) and Y for outcome (hypertension control). The X (community-based BP monitoring) significantly affects the M1 (hypertension treatment) and the Y (hypertension control). The M1 (hypertension treatment) significantly affects the Y (hypertension control). a1, b1 for indirect effects and c’ for direct effects

**Fig. 4:**
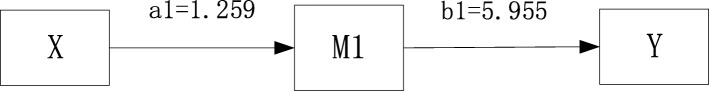
final mediation model of mediation analysis. X for predictor (community-based BP monitoring), M1 for mediator (medication treatment) and Y for outcome (hypertension control). The X (community-based BP monitoring) significantly affects the M1 (hypertension treatment) and the M1 significantly affects the Y (hypertension control). The direct effect (c’) of the X (community-based BP monitoring) to the Y (hypertension control) is insignificant when mediated by the M1 (hypertension treatment). a1 = 1.259, b1 = 5.955 for indirect effects, and a1b1 = 7.50 for the total effects. Sobel test t = 4.51, SE = 1.66, *P* < 0.001

As community-based BP monitoring service utilisation significantly predicted both the dependent variable of hypertension control and the mediator variable of hypertension treatment but not healthy lifestyle behaviours, the mediation model was adjusted based on our original hypothesis in Fig. 3. A final logistic regression was conducted including the mediator variable of hypertension treatment. The results in Table 5 showed the mediation effect of hypertension treatment on BP control. Taking hypertension treatment into consideration, BP control was significantly associated with hypertension treatment, but the independent variable of BP monitoring was not statistically significant anymore (*P* = 0.816). It indicated the use of BP monitoring service had no direct effect on BP control. The effect of independent variable BP monitoring on dependent variable BP control was completely mediated by the intermediate variable of hypertension treatment. Hypertension treatment played a perfect mediation role between monitoring and hypertension control. The Sobel test was conducted to further confirm its mediation effect (t = 4.51, SE = 1.66, *P* < 0.001). The final mediation model of the independent variable (community-based BP monitoring service utilisation), the mediator variable (medication treatment) and the dependent variable (hypertension control) was shown in Fig. 4.

**Table 5:** Binary logistic regression analysis for BP control associated with community-based BP monitoring mediated by hypertension treatment (Model 3), China Health and Retirement Longitudinal Study 2015

Variable	Category	%	β	*P*	OR	95% CI	
						Upper	Lower
Treatment				< 0.001**			
	No(ref)	11.96	1		1		
	Yes	88.04	5.955		385.786	53.437	2785.162
BP monitoring				0.816			
	No(ref)	67.89	1		1		
	Yes	32.11	0.022		1.023	0.846	1.237

*OR* = Odds Ratio. 95% CI = 95% Confidence interval for odds ratio

Adjusted for age, gender, educational attainment, household income, cohabitation status, residential region, working status, insurance status, BMI, and chronic diseases diagnosis. Wald F = 2.744, P < 0.001

**P < 0.01

## Discussion

Based on a nationally representative data from CHARLS, this study explored the association between utilisation of community-based BP monitoring service and BP control among hypertensive adults aged above 45. The prevalence of community-based BP monitoring service use was 32% among hypertensive persons in 2015. It showed an improvement in EPHS utilisation of hypertensive persons compared with a previous estimate in China (vs 8.1% in 2013) [32]. Compared with subjects whose BP was not monitored, those who used the community-based BP monitoring service had a higher likelihood of hypertension treatment as well as BP control but not healthy lifestyle behaviours. Moreover, better BP control was achieved through treatment improved by community-based BP monitoring.

Though improvement had been achieved over time, less than half of the hypertensive persons had their BP regularly examined by the community or village doctors. Percentage of hypertensive persons had their BP examined at least once annually was reported about 54–58% in 2011 and 51–65% in 2013 in China [32]. A similar finding was found in a community-based research in German that only 33.7% of hypertensive persons received at least twice BP examination in 2016 [35]. It indicated that the community-based service of hypertension management was underused. The prevalence of utilisation of hypertension monitoring service was still low.

This low reaching of community-based management may be due to several reasons. First, it is likely that many patients are unaware of this service in the community and consequently do not avail it [36]. Further implementation of this program should involve widespread public education to strengthen the residents’ consciousness of their health status and chronic disease management. Capability of community workforce should be further improved to gain residents’ trust. Second, the shortage in public health and primary care workforce may contribute to the lack of delivery in community-based services such as home visit BP examination and counselling [32]. Third, due to the relatively limited quality of care of grass-root health institutions, hypertensive persons who have multiple accesses to health care, for example, higher level hospitals in urban, may not choose to visit the community or village doctors.

On the other hand, utilisation of this service showed a development of hypertension management in grass-roots institutions, especially for the disadvantaged population [37]. Community-based hypertension monitoring was used more by patients with lower household income. The reason may be that it is free to have one’s BP examined by community or village doctors. The same result was found that more uneducated patients used this service. In spite of the underuse of BP monitoring, patients with higher educated level were more likely to control their BP. It is usually related to better health literacy for people who are educated [38]. It indicated that although community-based health management improved the health access for the disadvantaged population, its effectiveness could be further improved.

At the same time, this service has made a difference since an improvement of hypertension management was realized in less developed areas of China. In rural, patients used this service more than urban ones. Those patients from the western provinces where is the most underdeveloped area in China were mostly monitored and had controlled BP. It was possibly because of the local scarcity of multiple health facilities in these areas. It also takes longer to travel to higher level institutions from the rural [39]. It is no wonder that the community-based BP monitoring service has played a role in these regions. Nevertheless, as this service was not equally used, future studies should be focused on its geographic disparity.

Self or home monitoring of BP had been proved effective on increase hypertension treatment and BP control [40], whereas no national community-based monitoring program being evaluated yet for both economic and operative matters. This study presented that community-based BP monitoring once a season by health expertise had a significant impact on improving the rate of hypertension treatment, which was the key factor of hypertension control [41]. Having a usual source of care, optimizing adherence, and minimizing therapeutic inertia are associated with higher rates of BP control [42]. This EPHS covered programme made a difference in optimizing the prevention, recognition, and care of hypertension as a paradigm shift to population care and the use of strategies known to control BP. This finding indicates the broader international significance that with affordable funding of about 6 USD (40RMB) per capita, utilisation of free BP monitor provided by grass-roots’ expertise termly shall be a possible management tool to achieve better BP control at the population level. However, it is possible that people who are motivated to participate in monitoring are also motivated to manage their BP. If so causality may, in part, be attributed to their general motivation, rather than the intervention on its own. A future longitudinal study should be considered as it is probably impossible to ascertain this effect in this cross-sectional study.

Meanwhile, Studies had shown intervention program ran by grass-root institutions could effectively achieve higher BP control rate by improving not only medication use but also healthy lifestyle behaviours [43]. But a related study in the US presented regular access to primary care did not result in improved clinical outcomes because of its overall high prevalence of risk factors such as smoking and obesity [44]. The similar result was found in this study that the national utilisation of BP monitoring service in China was not related to health lifestyle behaviours such as alcohol intake, physical activity, and smoking. It is possible that community or village doctors paid more attention to patient’s medication use rather than on instructions on healthy lifestyle behaviours about drinking, smoking and physical activity. Also, compared with clinical intervention, it could take much longer and a harder time to encourage patients to achieve normal BP by establishing a healthy lifestyle. BP examination in 4 follow-up visits a year provided by national EPHS may not provide efficient education about healthy lifestyles. Further prospective studies should focus on healthy lifestyles establishment, as well as prescription patterns [45] and medication adherence [46, 47] of hypertensive persons receiving community-based management.

### Limitations

This study has four limitations. Firstly, hypertension diagnosis was not done by 24-h ambulatory blood pressure monitoring, which could be the best diagnosis way but would take a long time and not be cost-inefficient in a national survey [48]. Secondly, the status of medication use and community BP examination were not verified from the medical record, and therefore subject to recall bias. Thirdly, missing-values was checked in the biomarker data and in the information of community-based BP examination before analysis. So sample selection bias could be caused. At last, the causality effect of BP monitoring on BP control is limited due to the cross-sectional research design.

## Conclusion

The use of BP monitoring service had no direct effect on BP control. Utilisation of national community-based BP monitoring service is positively associated with BP control through a full medication effect of hypertension treatment. This study emphasized that through improving antihypertensive medication use among hypertensive persons, the national community-based BP monitoring could be an effective way to achieve hypertension control. Termly BP monitoring by community-based health expertise among hypertensive persons, for instance, once a season, can be recommended to public health policymakers for BP control through instructions on medication treatment and health behaviours. It was suggested that awareness of EPHS service and primary health care workforce should be enhanced to increase utilisation of community-based BP management. Education about healthy lifestyle behaviours in EPHS should be bolstered in the future.

## Data Availability

The datasets analysed during the current study are available on http://charls.pku.edu.cn/en/page/data/2015-charls-wave4.

## Abbreviations

BMI: Body mass index
BP: Blood pressure
CHARLS: China Health and Retirement Longitudinal Study
CVD: cardiovascular diseases
DBP: Diastolic blood pressure
EPHS: Essential public health service
IRB: Ethical Review Committee
NCD: Non-communicable diseases
OR: Odds ratio
SBP: Systolic blood pressure
